# The local importance of global infectious diseases

**DOI:** 10.1186/s40794-015-0004-7

**Published:** 2015-07-31

**Authors:** David L. Blazes, Mark S. Riddle, Edward T. Ryan

**Affiliations:** 1 Uniformed Services University of the Health Sciences, Bethesda, MD USA; 2 Naval Medical Research Center, Silver Spring, MD USA; 3 Harvard University, Boston, MA USA

Dengue in Tokyo and Key West. Leishmaniasis in Madrid and Texas. Chikungunya in Ravenna and Puerto Rico. SARS in Toronto. Ebola in Dallas. And measles everywhere. Population growth, globalization, climate change and urbanization together are reshaping the context upon which we must consider the global spread of emerging and historically tropical infectious diseases and their interface with non-communicable diseases of transitioning societies.

We live on a crowded and interconnected planet, with a projected global human population of at least 9 billion by 2050 [1]. Public health advances such as safe water, adequate sanitation, antibiotics, vaccines and balanced nutrition programs have extended overall life expectancy, even in remote, under-developed settings. The resulting unprecedented population surge has contributed to numerous challenges that will increasingly serve as counter-balances to these public health advances and will synergize with other inter-related factors such globalization, climate change and urbanization to contribute to the spread of dangerous infectious diseases.

International travel and immigration increase each year, with more than 1 billion humans crossing international borders in 2013 alone [2]. Many cross to embrace economic opportunities or to escape war or disaster. Many also travel to visit family or friends, or for business, education or leisure purposes. Exotic, remote and dangerous locations—often lacking public health infrastructure—are increasingly common destinations for travelers as well as sources of immigrants to the developed world. This “smaller world” effect brings with it much good in terms of societal connectedness and economic stimulation, but also individual risk to the traveler and the opportunity for spread of disease upon return.

Our current age of globalization also involves the increasingly rapid and direct transportation of food and other products that can introduce or facilitate disease transmission, whether it be cyclosporiasis associated with Latin America raspberries, [3] enterohemorrhagic *E. coli* infection associated with Middle Eastern fenugreek sprouts, [4] monkeypox from pet Gambian rats and prairie dogs, [5] or introduction into the US of *Aedes albopictus* (the Asian Tiger mosquito and potential vector of dengue and chikungunya) via imported used-tires from Asia [6].

Evolving global climate change and severe weather events can also be expected to have significant impacts on the distribution, spread and burden of many tropical and global infections; unfortunately, we just do not yet have the ability to accurately predict what these impacts will be. Two obvious areas that warrant special surveillance are vector-borne and water-borne diseases. For instance, regional burdens of malaria, dengue and chikungunya may all increase with the effect of increasing temperature on their mosquito vectors, and cholera and leptospirosis can increase in the setting of flooding associated with severe weather events [7]. The effect of severe weather on fragile health infrastructures will further undermine detection and control efforts, facilitating spread of pathogens. As the climate changes, unpredictable outcomes are also very likely, as humans alter their behavior in the setting of changing ecosystems, potentially increasing the likelihood of introducing new zoonotic infections into human populations.

The urbanization of the human population is impacting the risk of transmission of many infectious agents. Currently, more than 80 % of humans live in developing countries, and more than half of these people now live in urban settings, many in informal settlement or slum conditions (esa.un.org/unpd/wup/FinalReport/WUP2014.pdf Accessed 12 June 2015). This dense concentration of people lacking appropriate infrastructure support facilitates the transmission of contagious diseases while providing a ready source of susceptible individuals, whether it be influenza, typhoid fever or Ebola.

Very real progress has been made in bending the curve of infant and child mortality, though there is still a long way to go. The anticipated health, demographic and economic dividends of such an achievement has observed and anticipated consequences. However, with resultant increased life expectancy, the rise in cardiovascular and other chronic diseases represents a double burden. Scarce dollars will need to be effectively managed to address all population health issues.

We must face the reality that emerging infections are the norm and infections previously confined to one geographic location are now often globally relevant. Non-communicable diseases are extremely important, but we need not lose sight of the fact that infectious diseases still account for significant mortality and morbidity. Projections suggest that over the next few decades, infectious diseases will still account for one of five deaths globally [8]. Vaccines have been a major advancement with millions of deaths prevented each year by vaccination. However, challenges lay ahead in expanding the availability of existing vaccines to those in most need, development of new vaccines for malaria, HIV, dengue and enteric pathogens, and a dwindling capacity to add more vaccines to programs of immunization. Eradication (such as with current polio efforts) may alleviate the latter issue, though better delivery and program management science is needed to achieve more timely and cost-effective success. There are, however, many infectious disease challenges for which vaccines are not likely to be solutions, such as less common neglected tropical infections, the misuse of antimicrobials agents, the emergence and spread of multi-drug resistant organisms, and the rate of nosocomial infections. These all suggest that, even in resource-rich environments, we still need to make significant investments in combating infectious pathogens through advancements in policy.

Today, everyone lives internationally— even if they themselves have never left the community where they were born. No one knows what the future will hold, but recent and daily events strongly suggest that we will need to be adaptable to meet the emerging and evolving infectious challenges that lie ahead.

The journal of *Tropical Diseases, Travel Medicine and Vaccines* (*TDTMV*) was created with the hope of providing a forum where important discovery, observation and insights may find a global audience. As an open access journal, *TDTMV* articles will be freely available, and through an article-processing charge waiver program for researchers from low and low-middle income countries (as defined by the World Bank in September 2014) [9], we hope that this journal provides a forum for all, independent of ones means. The journal’s scope covers a broad range of topics, and in particular, will provide a forum for underserved topics such as chronic diseases in the developing world and migration medicine. Furthermore, *TDTMV* will endeavor to provide a much-needed forum for targeted updates in clinical tropical and travel medicine to serve as an easily-accessible source for the busy practitioner and trainee.

Finally, the launch and future success of this journal is dependent upon the esteemed Editorial Board, the peer reviewers and you. Thus, we welcome you to submit your research, observations, experience and perspectives to *Tropical Diseases, Travel Medicine and Vaccines*, not only to help the journal become a top journal in its field, but also improve the lives of many through shared knowledge. Our many thanks in advance.
